# [(*R*)-2,2-Bis(diphenyl­phosphan­yl)-1,1′-binaphthyl-κ^2^ *P*,*P*′]{2-[(2*R*)-1,2-diamino-1-(4-meth­oxy­phen­yl)-3-methyl­but­yl]-5-meth­oxy­phenyl-κ*C* ^1^}hydrido­ruthenium(II) benzene monosolvate

**DOI:** 10.1107/S1600536812046065

**Published:** 2012-11-17

**Authors:** Kamaluddin Abdur-Rashid, Alan J. Lough

**Affiliations:** a Kanata Chemical Technologies Inc., 101 College Street, Office 230, Toronto, Ontario, Canada M5G 1L7; b Department of Chemistry, University of Toronto, Toronto, Ontario, Canada M5S 3H6

## Abstract

In the title complex, [Ru(C_19_H_25_N_2_O_2_)H(C_44_H_32_P_2_)]·C_6_H_6_, the Ru^II^ ion is in a distorted octa­hedral coordination environment with the hydride H atom *trans* to the tertiary carbinamine N atom, giving an H—Ru—N angle of 160.8 (12)°. The equatorial sites are occupied by two P atoms, the secondary carbinamine N atom and a coordinated C atom.

## Related literature

For the synthesis of Ru(II) hydride complexes with an RuN_2_P_2_ coordination environment, see: Abdur-Rashid, Faatz *et al.* (2001 ▶); Abdur-Rashid, Abbel *et al.* (2005 ▶); Ohkuma *et al.* (1995 ▶). For their use as catalysts, see: Abdur-Rashid, Guo *et al.* (2005 ▶); Abdur-Rashid *et al.* (2000 ▶); Abdur-Rashid, Lough *et al.* (2001 ▶); Cobley & Henschke (2003 ▶); Doucet *et al.* (1998 ▶); Matsumura *et al.* (2011 ▶). For related structures, see: Guo *et al.* (2004 ▶); Li *et al.* (2004 ▶). For kinetic studies, see: Abbel *et al.* (2005 ▶); Abdur-Rashid *et al.* (2002 ▶). 
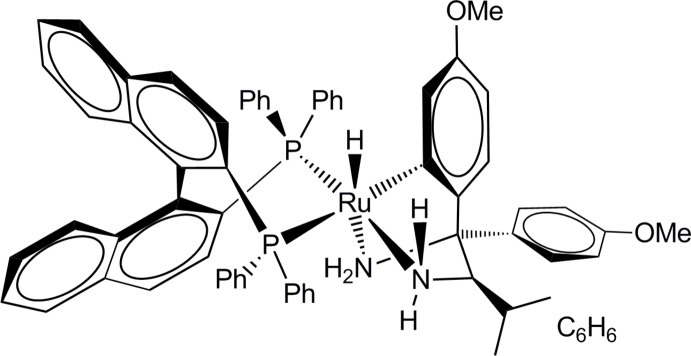


## Experimental

### 

#### Crystal data

[Ru(C_19_H_25_N_2_O_2_)H(C_44_H_32_P_2_)]·C_6_H_6_
*M* *_r_* = 1116.23
Monoclinic, 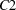

*a* = 38.8848 (8) Å
*b* = 13.5741 (3) Å
*c* = 10.8871 (2) Å
β = 103.967 (1)°
*V* = 5576.6 (2) Å^3^
*Z* = 4
Mo *K*α radiation
μ = 0.39 mm^−1^
*T* = 150 K
0.35 × 0.32 × 0.25 mm

#### Data collection

Nonius KappaCCD diffractometer
Absorption correction: multi-scan (*SORTAV*; Blessing, 1995 ▶) *T*_min_ = 0.876, *T*_max_ = 0.909
21246 measured reflections
11534 independent reflections
10079 reflections with *I* > 2σ(*I*)
*R*_int_ = 0.051

#### Refinement

*R*[*F* ^2^ > 2σ(*F* ^2^)] = 0.039
*wR*(*F* ^2^) = 0.089
*S* = 1.04
11534 reflections
698 parameters
1 restraint
H atoms treated by a mixture of independent and constrained refinement
Δρ_max_ = 0.54 e Å^−3^
Δρ_min_ = −0.54 e Å^−3^
Absolute structure: Flack (1983 ▶), 4924 Friedel pairs
Flack parameter: −0.045 (19)

### 

Data collection: *COLLECT* (Nonius, 2002 ▶); cell refinement: *DENZO-SMN* (Otwinowski & Minor, 1997 ▶); data reduction: *DENZO-SMN*; program(s) used to solve structure: *SIR92* (Altomare *et al.*, 1994 ▶); program(s) used to refine structure: *SHELXTL* (Sheldrick, 2008 ▶); molecular graphics: *SHELXTL*; software used to prepare material for publication: *SHELXTL*.

## Supplementary Material

Crystal structure: contains datablock(s) global, I. DOI: 10.1107/S1600536812046065/vm2180sup1.cif

Structure factors: contains datablock(s) I. DOI: 10.1107/S1600536812046065/vm2180Isup2.hkl

Additional supplementary materials:  crystallographic information; 3D view; checkCIF report

## Figures and Tables

**Fig. 1.**
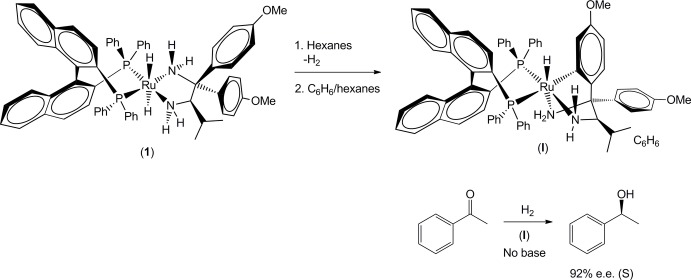
The synthetic scheme

**Fig. 2.**
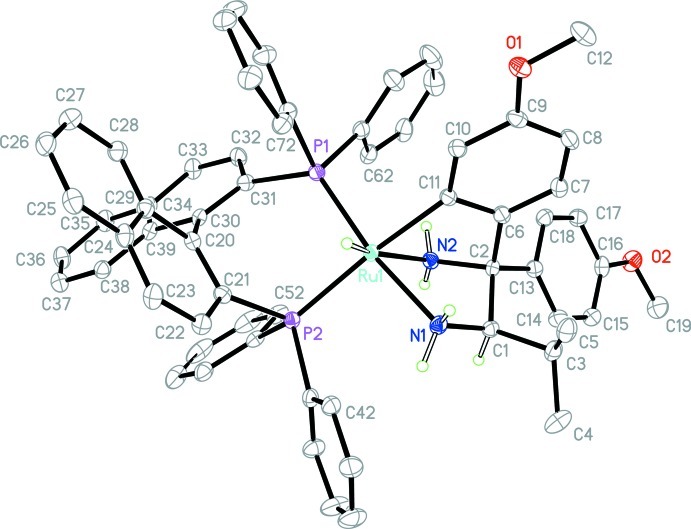
The molecular structure of (I). Displacement ellipsoids are at the 30% probability level. The benzene solvent molecule and H atoms bonded to C atoms are not shown.

## supplementary crystallographic information

### Comment

Asymmetric hydrogenation of ketones and imines constitute a very valuable technology for the production of chiral alcohols and amines which are useful precursors and end products in the pharmaceutical, agrochemical, fragrance and flavor industries. A variety of useful catalysts have been developed that are broadly applicable, very active and highly selective. These include ruthenium complexes of the type RuCl_2_(diphosphine)(diamine) originally developed by Noyori and coworkers for ketone hydrogenation processes (Ohkuma *et al.*, 1995; Doucet *et al.*, 1998). Our research (Abdur-Rashid *et al.*, 2000; Abdur-Rashid, Lough *et al.*, 2001) and that of others (Cobley & Henschke, 2003) demonstrated that these catalysts were also capable of reducing imines to amines, including the production of single isomer chiral products. There have been extensive investigations towards isolating and understanding the nature of the active catalytic species generated from RuCl_2_(diphosphine)(diamine) under the reaction conditions of added base in the presence of hydrogen gas. Our seminal papers (Abdur-Rashid, Faatz *et al.*, 2001; Abdur-Rashid *et al.*, 2002; Abbel *et al.*, 2005) proved that the active catalysts are ruthenium dihydride compounds and demonstrated the role of the bifunctional *cis*-Ru—H···H—N motif to provide nascent, polarized dihydrogen (H^δ^^+^···H^δ^^-^) for the catalytic ionic hydrogenation of polar C═O and C═N bonds. Our research also resulted in the unprecedented isolation and characterization of stable hydridoamido intermediates and proved that these species are responsible for the rapid heterolytic activation and splitting of hydrogen gas (Abdur-Rashid, Faatz *et al.*, 2001; Abdur-Rashid, *et al.*, 2002). Recently Ohkuma and coworkers reported chlororuthenabicyclic compounds (Matsumura *et al.*, 2011) which are derived from ruthenium complexes containing a chiral diphosphine and the chiral diamine ligand daipen. The work demonstrated that in the presence of base and hydrogen gas these ruthenabicyclic compounds are very useful for the hydrogenation of a wide variety of ketones and are capable of producing chiral alcohols with high enantioselectivities. The authors proposed a mechanism based on a hydridoruthenabicyclic catalytic species. However, their attempts to isolate and characterize this species were not successful. We previously reported the preparation and characterization of the *trans*-dihydride complex RuH_2_(*R*-binap)(*R*-daipen) (1) (Fig. 1), [*R*-Binap = (*R*)-bis(diphenylphosphanyl)-1,1-binaphthyl and *R*-Daipen = (*R*)-1,1-bis(4-methoxyphenyl)-3-methylbutane-1,2-diamine] and proved that this compound was an active base-free catalyst for the hydrogenation of ketones in the presence of hydrogen gas (Abdur-Rashid, Faatz *et al.*, 2001).

The title compound (I) was readily obtained from (1) by the loss of hydrogen upon stirring a suspension of (1) in hexanes for 48 h at room temperature under an atmosphere of argon. It was demonstrated that the novel hydridoruthenabicyclic compound (I) is also a very effective base-free catalyst for the hydrogenation of ketones to alcohols. For example, a catalytic amount of (I) resulted in the complete conversion of neat acetophenone (S:*C* = 2000:1) to (S)-1-phenylethanol (92% e.e.) within 12 h at room temperature in the presence of hydrogen gas (3 atm.).

The molecular structure of complex (I) is shown in Fig. 2. The Ru^II^ ion is in a distorted octahedral coordination environment with the hydride H atom (H1RU) *trans* to the tertiary carbinamine nitrogen atom (N2) giving an H1RU—Ru1—N2 angle of 160.8 (12)°. The equatorial sites are occupied by two phosphorus atoms (P1 and P2), the secondary carbinamine nitrogen atom (N1) and a coordinated carbon atom (C11). The geometric parameters in (I) are comparable to related structures (Guo *et al.*, 2004; Li *et al.*, 2004).

### Experimental

A solution of K-Selectride (100 ml of a 1.0 *M* solution in THF) was added to a solution of RuHCl(*R*-binap)(*R*-daipen) (100 mg) in THF (5 ml) and the mixture stirred under hydrogen gas at room temperature for 2 h. The mixture was filtered and the filtrate evaporated to dryness to give the bright yellow dihydride compound RuH_2_(*R*-binap)(*R*-daipen) (1) [*R*-Binap = (*R*)-bis(diphenylphosphanyl)-1,1-binaphthyl and *R*-Daipen = (*R*)-1,1-Bis(4-methoxyphenyl)-3-methylbutane-1,2-diamine]. This was suspended in hexanes (5 ml) and the suspension stirred for 48 h at room temperature under argon. The solids were filtered and washed with hexanes (5 ml) and dried under vacuum to give 76 mg of the hydridoruthenabicyclic compound (I) as a bright yellow solid. X-ray diffraction quality single crystals were grown by the slow diffusion of hexanes into a solution of (I) in benzene.

### Refinement

Hydrogen atoms bonded to C atoms were placed in calculated positions with C—H = 0.95–1.00 Å and were included in the refinement in a riding-model approximation with *U*_iso_(H) = 1.2*U*_eq_(C) or 1.5*U*_eq_(C) for methyl C atoms. The hydride and amine H atoms were refined independently with isotropic displacement parameters. The benzene solvent molecule was fitted as a regular hexagon with C—C = 1.39 Å using the AFIX 66 command in *SHELXL* (Sheldrick, 2008).

### Crystal data

**Table:**

[Ru(C_19_H_25_N_2_O_2_)H(C_44_H_32_P_2_)]·C_6_H_6_	*F*(000) = 2328
*M**_r_* = 1116.23	*D*_x_ = 1.330 Mg m^−^^3^
Monoclinic, *C*2	Mo *K*α radiation, λ = 0.71073 Å
Hall symbol: C 2y	Cell parameters from 11534 reflections
*a* = 38.8848 (8) Å	θ = 2.7–27.5°
*b* = 13.5741 (3) Å	µ = 0.39 mm^−^^1^
*c* = 10.8871 (2) Å	*T* = 150 K
β = 103.967 (1)°	Block, yellow
*V* = 5576.6 (2) Å^3^	0.35 × 0.32 × 0.25 mm
*Z* = 4	

### Data collection

**Table:**

Nonius KappaCCD diffractometer	11534 independent reflections
Radiation source: fine-focus sealed tube	10079 reflections with *I* > 2σ(*I*)
Graphite monochromator	*R*_int_ = 0.051
Detector resolution: 9 pixels mm^-1^	θ_max_ = 27.5°, θ_min_ = 2.7°
φ scans and ω scans with κ offsets	*h* = −50→50
Absorption correction: multi-scan (*SORTAV*; Blessing, 1995)	*k* = −17→17
*T*_min_ = 0.876, *T*_max_ = 0.909	*l* = −14→14
21246 measured reflections	

### Refinement

**Table:**

Refinement on *F*^2^	Hydrogen site location: inferred from neighbouring sites
Least-squares matrix: full	H atoms treated by a mixture of independent and constrained refinement
*R*[*F*^2^ > 2σ(*F*^2^)] = 0.039	*w* = 1/[σ^2^(*F*_o_^2^) + (0.0286*P*)^2^ + 2.2793*P*] where *P* = (*F*_o_^2^ + 2*F*_c_^2^)/3
*wR*(*F*^2^) = 0.089	(Δ/σ)_max_ = 0.002
*S* = 1.04	Δρ_max_ = 0.54 e Å^−^^3^
11534 reflections	Δρ_min_ = −0.54 e Å^−^^3^
698 parameters	Extinction correction: *SHELXTL* (Sheldrick, 2008), Fc^*^=kFc[1+0.001xFc^2^λ^3^/sin(2θ)]^-1/4^
1 restraint	Extinction coefficient: 0.00045 (11)
Primary atom site location: structure-invariant direct methods	Absolute structure: Flack (1983), 4924 Friedel pairs
Secondary atom site location: difference Fourier map	Flack parameter: −0.045 (19)

### Special details

**Table:**

Geometry. All e.s.d.'s (except the e.s.d. in the dihedral angle between two l.s. planes) are estimated using the full covariance matrix. The cell e.s.d.'s are taken into account individually in the estimation of e.s.d.'s in distances, angles and torsion angles; correlations between e.s.d.'s in cell parameters are only used when they are defined by crystal symmetry. An approximate (isotropic) treatment of cell e.s.d.'s is used for estimating e.s.d.'s involving l.s. planes.
Refinement. Refinement of *F*^2^ against ALL reflections. The weighted *R*-factor *wR* and goodness of fit *S* are based on *F*^2^, conventional *R*-factors *R* are based on *F*, with *F* set to zero for negative *F*^2^. The threshold expression of *F*^2^ > σ(*F*^2^) is used only for calculating *R*-factors(gt) *etc*. and is not relevant to the choice of reflections for refinement. *R*-factors based on *F*^2^ are statistically about twice as large as those based on *F*, and *R*- factors based on ALL data will be even larger.

### Fractional atomic coordinates and isotropic or equivalent isotropic displacement parameters (Å^2^)

**Table:**

	*x*	*y*	*z*	*U*_iso_*/*U*_eq_	
Ru1	0.365924 (6)	0.782933 (18)	0.603130 (19)	0.02226 (7)	
H1RU	0.3380 (8)	0.809 (3)	0.685 (3)	0.029 (9)*	
P1	0.40933 (2)	0.81338 (6)	0.77366 (7)	0.02362 (18)	
P2	0.35821 (2)	0.94374 (6)	0.54146 (7)	0.02201 (17)	
O1	0.33911 (8)	0.4662 (2)	0.8879 (2)	0.0368 (7)	
O2	0.44751 (7)	0.3603 (2)	0.1801 (2)	0.0398 (6)	
N1	0.32414 (7)	0.7297 (2)	0.4435 (3)	0.0275 (6)	
H1B	0.3095 (10)	0.778 (4)	0.392 (4)	0.056 (11)*	
H1C	0.3104 (12)	0.705 (3)	0.489 (4)	0.048 (13)*	
N2	0.39494 (7)	0.6998 (2)	0.4842 (3)	0.0247 (6)	
H2A	0.4178 (11)	0.684 (3)	0.534 (3)	0.039 (11)*	
H2B	0.3993 (9)	0.725 (3)	0.428 (3)	0.016 (9)*	
C1	0.33628 (8)	0.6555 (2)	0.3619 (3)	0.0254 (7)	
H1A	0.3421	0.6927	0.2903	0.031*	
C2	0.37185 (8)	0.6111 (2)	0.4411 (3)	0.0236 (6)	
C3	0.30748 (10)	0.5792 (3)	0.3020 (3)	0.0317 (8)	
H3A	0.3198	0.5172	0.2871	0.038*	
C4	0.28619 (11)	0.6170 (4)	0.1732 (4)	0.0482 (11)	
H4A	0.2681	0.5684	0.1354	0.072*	
H4B	0.3022	0.6272	0.1172	0.072*	
H4C	0.2747	0.6794	0.1848	0.072*	
C5	0.28160 (9)	0.5531 (3)	0.3852 (3)	0.0368 (8)	
H5A	0.2951	0.5361	0.4707	0.055*	
H5B	0.2669	0.4969	0.3481	0.055*	
H5C	0.2664	0.6099	0.3894	0.055*	
C6	0.36574 (8)	0.5661 (3)	0.5624 (3)	0.0260 (7)	
C7	0.36063 (8)	0.4660 (3)	0.5759 (3)	0.0279 (7)	
H7A	0.3635	0.4223	0.5109	0.033*	
C8	0.35141 (9)	0.4281 (3)	0.6828 (3)	0.0315 (7)	
H8A	0.3475	0.3595	0.6909	0.038*	
C9	0.34817 (9)	0.4944 (3)	0.7772 (3)	0.0294 (7)	
C10	0.35333 (9)	0.5946 (2)	0.7629 (3)	0.0279 (7)	
H10A	0.3507	0.6378	0.8288	0.033*	
C11	0.36215 (8)	0.6352 (2)	0.6568 (3)	0.0237 (6)	
C12	0.33415 (11)	0.3639 (3)	0.9057 (4)	0.0443 (10)	
H12A	0.3284	0.3535	0.9876	0.066*	
H12B	0.3559	0.3282	0.9039	0.066*	
H12C	0.3147	0.3394	0.8378	0.066*	
C13	0.39027 (8)	0.5435 (2)	0.3657 (3)	0.0266 (7)	
C14	0.38130 (9)	0.5383 (3)	0.2346 (3)	0.0315 (8)	
H14A	0.3621	0.5770	0.1888	0.038*	
C15	0.39949 (9)	0.4781 (3)	0.1678 (3)	0.0325 (8)	
H15A	0.3926	0.4755	0.0780	0.039*	
C16	0.42779 (9)	0.4219 (3)	0.2338 (3)	0.0320 (7)	
C17	0.43839 (9)	0.4296 (3)	0.3658 (3)	0.0320 (8)	
H17A	0.4584	0.3936	0.4113	0.038*	
C18	0.41996 (9)	0.4893 (3)	0.4300 (3)	0.0308 (7)	
H18A	0.4275	0.4939	0.5196	0.037*	
C19	0.43718 (12)	0.3472 (3)	0.0455 (4)	0.0470 (10)	
H19A	0.4545	0.3050	0.0188	0.071*	
H19B	0.4363	0.4114	0.0038	0.071*	
H19C	0.4137	0.3163	0.0221	0.071*	
C20	0.37929 (8)	1.0340 (2)	0.7784 (3)	0.0242 (6)	
C21	0.35121 (8)	1.0207 (2)	0.6733 (3)	0.0249 (7)	
C22	0.31719 (8)	1.0571 (3)	0.6782 (3)	0.0287 (7)	
H22A	0.2982	1.0527	0.6048	0.034*	
C23	0.31115 (9)	1.0981 (3)	0.7854 (3)	0.0328 (8)	
H23A	0.2882	1.1226	0.7848	0.039*	
C24	0.33835 (10)	1.1045 (3)	0.8968 (3)	0.0298 (8)	
C25	0.33263 (10)	1.1434 (3)	1.0118 (3)	0.0346 (8)	
H25A	0.3095	1.1645	1.0145	0.042*	
C26	0.35911 (11)	1.1509 (3)	1.1161 (3)	0.0403 (9)	
H26A	0.3548	1.1785	1.1912	0.048*	
C27	0.39342 (11)	1.1178 (3)	1.1146 (3)	0.0414 (9)	
H27A	0.4119	1.1215	1.1895	0.050*	
C28	0.40020 (10)	1.0803 (3)	1.0063 (3)	0.0322 (8)	
H28A	0.4235	1.0592	1.0065	0.039*	
C29	0.37304 (9)	1.0725 (2)	0.8940 (3)	0.0270 (7)	
C30	0.41747 (8)	1.0206 (2)	0.7768 (3)	0.0227 (6)	
C31	0.43490 (8)	0.9308 (3)	0.7890 (3)	0.0250 (7)	
C32	0.47271 (9)	0.9318 (3)	0.8113 (3)	0.0300 (7)	
H32A	0.4853	0.8714	0.8274	0.036*	
C33	0.49113 (9)	1.0167 (3)	0.8102 (3)	0.0306 (8)	
H33A	0.5162	1.0143	0.8253	0.037*	
C34	0.47370 (8)	1.1086 (3)	0.7871 (3)	0.0275 (7)	
C35	0.49190 (9)	1.1972 (3)	0.7781 (3)	0.0342 (8)	
H35A	0.5168	1.1957	0.7875	0.041*	
C36	0.47426 (8)	1.2858 (4)	0.7560 (3)	0.0349 (7)	
H36A	0.4868	1.3447	0.7487	0.042*	
C37	0.43762 (8)	1.2884 (3)	0.7444 (3)	0.0327 (7)	
H37A	0.4254	1.3495	0.7301	0.039*	
C38	0.41919 (9)	1.2040 (2)	0.7533 (3)	0.0272 (7)	
H38A	0.3944	1.2077	0.7459	0.033*	
C39	0.43620 (8)	1.1113 (2)	0.7731 (3)	0.0250 (7)	
C41	0.31730 (8)	0.9610 (2)	0.4156 (3)	0.0256 (7)	
C42	0.28469 (9)	0.9283 (3)	0.4369 (3)	0.0295 (7)	
H42A	0.2840	0.9022	0.5172	0.035*	
C43	0.25372 (9)	0.9338 (3)	0.3420 (3)	0.0384 (8)	
H43A	0.2318	0.9140	0.3585	0.046*	
C44	0.25466 (10)	0.9684 (3)	0.2222 (4)	0.0493 (11)	
H44A	0.2335	0.9732	0.1572	0.059*	
C45	0.28661 (11)	0.9954 (4)	0.1995 (4)	0.0485 (11)	
H45A	0.2876	1.0158	0.1169	0.058*	
C46	0.31755 (10)	0.9933 (3)	0.2953 (3)	0.0347 (9)	
H46A	0.3392	1.0144	0.2779	0.042*	
C51	0.39023 (8)	1.0214 (3)	0.4854 (3)	0.0270 (7)	
C52	0.42172 (9)	0.9793 (3)	0.4729 (3)	0.0354 (8)	
H52A	0.4252	0.9102	0.4825	0.042*	
C53	0.44850 (10)	1.0390 (4)	0.4461 (3)	0.0517 (12)	
H53A	0.4700	1.0101	0.4371	0.062*	
C54	0.44376 (12)	1.1387 (4)	0.4328 (4)	0.0570 (14)	
H54A	0.4623	1.1787	0.4168	0.068*	
C55	0.41235 (13)	1.1818 (3)	0.4424 (3)	0.0487 (11)	
H55A	0.4089	1.2508	0.4311	0.058*	
C56	0.38568 (10)	1.1226 (3)	0.4688 (3)	0.0348 (8)	
H56A	0.3640	1.1520	0.4756	0.042*	
C61	0.44425 (9)	0.7199 (3)	0.7828 (3)	0.0303 (7)	
C62	0.46595 (9)	0.7245 (3)	0.6967 (3)	0.0332 (8)	
H62A	0.4641	0.7800	0.6424	0.040*	
C63	0.48997 (10)	0.6504 (3)	0.6887 (4)	0.0408 (9)	
H63A	0.5047	0.6554	0.6308	0.049*	
C64	0.49206 (12)	0.5694 (3)	0.7665 (5)	0.0534 (12)	
H64A	0.5086	0.5185	0.7634	0.064*	
C65	0.47035 (12)	0.5619 (3)	0.8486 (4)	0.0543 (11)	
H65A	0.4714	0.5046	0.8995	0.065*	
C66	0.44688 (10)	0.6369 (3)	0.8581 (4)	0.0406 (9)	
H66A	0.4325	0.6312	0.9170	0.049*	
C71	0.40170 (9)	0.8134 (2)	0.9349 (3)	0.0298 (7)	
C72	0.36758 (9)	0.8250 (3)	0.9506 (3)	0.0327 (7)	
H72A	0.3480	0.8238	0.8788	0.039*	
C73	0.36170 (11)	0.8386 (3)	1.0712 (3)	0.0396 (9)	
H73A	0.3382	0.8465	1.0806	0.048*	
C74	0.38965 (12)	0.8404 (3)	1.1762 (3)	0.0429 (10)	
H74A	0.3856	0.8518	1.2576	0.052*	
C75	0.42389 (12)	0.8256 (3)	1.1626 (3)	0.0474 (10)	
H75A	0.4431	0.8239	1.2354	0.057*	
C76	0.43013 (10)	0.8134 (3)	1.0431 (3)	0.0386 (9)	
H76A	0.4537	0.8049	1.0344	0.046*	
C1S	0.23578 (10)	0.2324 (4)	0.2839 (5)	0.113 (3)	
H1SA	0.2148	0.2043	0.2993	0.135*	
C2S	0.23558 (14)	0.2706 (5)	0.1653 (4)	0.141 (4)	
H2SA	0.2145	0.2686	0.0996	0.170*	
C3S	0.2662 (2)	0.3117 (4)	0.1429 (4)	0.134 (4)	
H3SA	0.2661	0.3378	0.0619	0.160*	
C4S	0.29709 (15)	0.3146 (3)	0.2392 (7)	0.119 (3)	
H4SA	0.3180	0.3427	0.2239	0.143*	
C5S	0.29729 (9)	0.2764 (4)	0.3578 (5)	0.101 (2)	
H5SA	0.3184	0.2784	0.4236	0.122*	
C6S	0.26664 (14)	0.2353 (3)	0.3802 (4)	0.0886 (18)	
H6SA	0.2668	0.2092	0.4613	0.106*	

### Atomic displacement parameters (Å^2^)

**Table:**

	*U*^11^	*U*^22^	*U*^33^	*U*^12^	*U*^13^	*U*^23^
Ru1	0.01979 (11)	0.02082 (11)	0.02517 (11)	−0.00075 (12)	0.00348 (8)	−0.00106 (12)
P1	0.0211 (4)	0.0227 (4)	0.0256 (4)	0.0000 (3)	0.0029 (3)	−0.0001 (3)
P2	0.0188 (4)	0.0225 (4)	0.0243 (4)	−0.0010 (3)	0.0042 (3)	−0.0011 (3)
O1	0.0467 (17)	0.0315 (16)	0.0327 (14)	−0.0044 (13)	0.0104 (12)	0.0072 (12)
O2	0.0353 (14)	0.0433 (15)	0.0419 (14)	0.0072 (12)	0.0116 (12)	−0.0109 (11)
N1	0.0214 (14)	0.0269 (16)	0.0313 (14)	0.0012 (12)	0.0005 (12)	−0.0045 (12)
N2	0.0241 (15)	0.0223 (15)	0.0274 (14)	−0.0010 (12)	0.0057 (12)	0.0008 (12)
C1	0.0222 (15)	0.0253 (17)	0.0267 (15)	0.0007 (13)	0.0018 (12)	−0.0021 (13)
C2	0.0245 (16)	0.0203 (16)	0.0246 (15)	−0.0034 (13)	0.0029 (12)	−0.0023 (12)
C3	0.0270 (19)	0.0313 (19)	0.0344 (18)	−0.0028 (16)	0.0027 (14)	−0.0073 (15)
C4	0.034 (2)	0.072 (3)	0.034 (2)	−0.008 (2)	−0.0001 (16)	−0.008 (2)
C5	0.0269 (18)	0.038 (2)	0.0444 (19)	−0.0074 (16)	0.0065 (15)	−0.0074 (16)
C6	0.0210 (16)	0.0236 (17)	0.0317 (16)	−0.0005 (14)	0.0028 (13)	0.0007 (14)
C7	0.0231 (16)	0.0248 (17)	0.0351 (17)	0.0013 (14)	0.0060 (13)	−0.0031 (14)
C8	0.0289 (18)	0.0214 (17)	0.0419 (18)	−0.0012 (14)	0.0039 (14)	0.0054 (15)
C9	0.0239 (17)	0.0295 (18)	0.0330 (17)	0.0019 (14)	0.0031 (13)	0.0090 (14)
C10	0.0274 (17)	0.0253 (18)	0.0288 (16)	−0.0030 (14)	0.0029 (13)	0.0022 (13)
C11	0.0204 (15)	0.0226 (16)	0.0269 (15)	−0.0017 (13)	0.0031 (12)	0.0026 (13)
C12	0.049 (2)	0.034 (2)	0.052 (2)	−0.0016 (19)	0.017 (2)	0.0153 (18)
C13	0.0233 (16)	0.0259 (17)	0.0305 (16)	0.0002 (14)	0.0059 (12)	−0.0012 (13)
C14	0.0284 (18)	0.036 (2)	0.0293 (16)	0.0030 (15)	0.0051 (13)	−0.0003 (15)
C15	0.0311 (19)	0.034 (2)	0.0316 (18)	−0.0008 (16)	0.0060 (15)	−0.0009 (15)
C16	0.0301 (18)	0.0269 (18)	0.0417 (18)	−0.0060 (15)	0.0137 (15)	−0.0072 (15)
C17	0.0264 (18)	0.033 (2)	0.0357 (18)	0.0039 (16)	0.0048 (14)	0.0006 (16)
C18	0.0291 (18)	0.0301 (18)	0.0307 (17)	0.0012 (15)	0.0025 (14)	−0.0027 (14)
C19	0.052 (2)	0.050 (3)	0.043 (2)	0.008 (2)	0.0173 (19)	−0.0104 (19)
C20	0.0245 (16)	0.0206 (16)	0.0281 (15)	0.0001 (13)	0.0077 (12)	−0.0001 (13)
C21	0.0231 (16)	0.0236 (17)	0.0284 (15)	0.0008 (14)	0.0067 (12)	0.0020 (13)
C22	0.0197 (16)	0.0310 (19)	0.0350 (17)	0.0024 (14)	0.0057 (13)	−0.0044 (14)
C23	0.0244 (17)	0.034 (2)	0.0435 (19)	0.0001 (15)	0.0141 (15)	−0.0099 (16)
C24	0.033 (2)	0.026 (2)	0.0345 (19)	−0.0032 (16)	0.0158 (16)	−0.0049 (15)
C25	0.038 (2)	0.030 (2)	0.042 (2)	0.0004 (16)	0.0214 (16)	−0.0015 (16)
C26	0.063 (3)	0.032 (2)	0.0300 (18)	0.0003 (18)	0.0198 (17)	−0.0032 (15)
C27	0.051 (2)	0.043 (2)	0.0293 (18)	0.001 (2)	0.0080 (17)	−0.0016 (17)
C28	0.0345 (19)	0.0322 (19)	0.0303 (16)	0.0040 (16)	0.0082 (14)	−0.0018 (14)
C29	0.0328 (18)	0.0225 (16)	0.0274 (15)	−0.0036 (14)	0.0102 (13)	0.0001 (13)
C30	0.0215 (15)	0.0239 (16)	0.0223 (14)	−0.0029 (13)	0.0047 (11)	−0.0049 (12)
C31	0.0231 (16)	0.0268 (18)	0.0250 (15)	−0.0019 (14)	0.0055 (12)	0.0014 (13)
C32	0.0231 (16)	0.0271 (18)	0.0384 (17)	0.0051 (14)	0.0046 (13)	−0.0030 (14)
C33	0.0170 (16)	0.0304 (19)	0.0440 (19)	−0.0010 (14)	0.0066 (14)	−0.0070 (16)
C34	0.0240 (16)	0.0285 (18)	0.0309 (16)	−0.0037 (14)	0.0081 (13)	−0.0062 (13)
C35	0.0257 (17)	0.034 (2)	0.0443 (19)	−0.0078 (15)	0.0114 (15)	−0.0054 (16)
C36	0.0359 (16)	0.0251 (15)	0.0465 (16)	−0.006 (2)	0.0157 (13)	−0.003 (2)
C37	0.0356 (16)	0.0276 (17)	0.0356 (14)	0.001 (2)	0.0096 (12)	−0.0017 (19)
C38	0.0261 (17)	0.0268 (18)	0.0278 (15)	−0.0022 (14)	0.0050 (13)	−0.0022 (13)
C39	0.0247 (16)	0.0262 (17)	0.0241 (15)	−0.0033 (14)	0.0061 (12)	−0.0070 (13)
C41	0.0217 (16)	0.0221 (16)	0.0323 (16)	−0.0023 (13)	0.0053 (13)	−0.0041 (13)
C42	0.0265 (17)	0.0284 (18)	0.0319 (16)	−0.0002 (15)	0.0036 (13)	−0.0019 (14)
C43	0.0257 (18)	0.040 (2)	0.046 (2)	0.0023 (17)	0.0025 (15)	0.0000 (17)
C44	0.031 (2)	0.063 (3)	0.044 (2)	−0.010 (2)	−0.0103 (16)	0.009 (2)
C45	0.038 (2)	0.068 (3)	0.033 (2)	−0.013 (2)	−0.0042 (16)	0.011 (2)
C46	0.0270 (19)	0.043 (2)	0.0316 (18)	−0.0062 (17)	0.0020 (15)	0.0018 (16)
C51	0.0241 (16)	0.0345 (19)	0.0206 (14)	−0.0043 (15)	0.0018 (12)	−0.0033 (13)
C52	0.0264 (18)	0.053 (2)	0.0267 (16)	−0.0031 (16)	0.0074 (13)	−0.0045 (15)
C53	0.029 (2)	0.097 (4)	0.0314 (19)	−0.015 (2)	0.0104 (15)	−0.008 (2)
C54	0.050 (3)	0.090 (4)	0.031 (2)	−0.050 (3)	0.0090 (18)	−0.005 (2)
C55	0.071 (3)	0.046 (2)	0.0299 (18)	−0.032 (2)	0.0137 (18)	−0.0001 (17)
C56	0.043 (2)	0.036 (2)	0.0254 (16)	−0.0092 (18)	0.0075 (15)	−0.0007 (15)
C61	0.0243 (17)	0.0277 (18)	0.0347 (17)	0.0008 (14)	−0.0011 (13)	−0.0042 (14)
C62	0.0271 (18)	0.033 (2)	0.0359 (18)	0.0049 (15)	0.0010 (14)	−0.0093 (15)
C63	0.0277 (18)	0.037 (2)	0.056 (2)	0.0038 (16)	0.0061 (16)	−0.0146 (18)
C64	0.034 (2)	0.041 (3)	0.083 (3)	0.008 (2)	0.009 (2)	−0.007 (2)
C65	0.047 (3)	0.033 (2)	0.079 (3)	0.009 (2)	0.007 (2)	0.011 (2)
C66	0.033 (2)	0.032 (2)	0.054 (2)	−0.0013 (17)	0.0036 (17)	0.0073 (18)
C71	0.0344 (17)	0.0259 (18)	0.0279 (15)	−0.0046 (14)	0.0053 (13)	0.0002 (12)
C72	0.0350 (19)	0.0342 (18)	0.0291 (16)	−0.0041 (15)	0.0084 (14)	−0.0033 (14)
C73	0.042 (2)	0.044 (2)	0.0365 (18)	−0.0026 (18)	0.0168 (16)	−0.0014 (17)
C74	0.063 (3)	0.041 (2)	0.0257 (18)	−0.007 (2)	0.0133 (17)	0.0020 (16)
C75	0.057 (3)	0.053 (2)	0.0252 (17)	−0.008 (2)	−0.0026 (16)	0.0030 (16)
C76	0.0342 (18)	0.046 (2)	0.0329 (16)	−0.0025 (16)	0.0026 (14)	0.0028 (15)
C1S	0.059 (4)	0.151 (7)	0.138 (6)	−0.013 (4)	0.044 (4)	−0.062 (5)
C2S	0.104 (6)	0.227 (11)	0.082 (4)	0.089 (7)	0.000 (4)	−0.020 (6)
C3S	0.199 (9)	0.117 (8)	0.111 (6)	0.067 (7)	0.090 (7)	0.006 (5)
C4S	0.128 (7)	0.055 (4)	0.215 (9)	−0.013 (4)	0.120 (7)	−0.041 (5)
C5S	0.063 (3)	0.070 (4)	0.158 (6)	0.010 (4)	0.000 (4)	−0.055 (5)
C6S	0.110 (5)	0.067 (4)	0.088 (4)	0.010 (4)	0.024 (4)	0.005 (3)

### Geometric parameters (Å, º)

**Table:**

Ru1—H1RU	1.60 (3)	C27—H27A	0.9500
Ru1—C11	2.104 (3)	C28—C29	1.413 (5)
Ru1—N1	2.195 (3)	C28—H28A	0.9500
Ru1—N2	2.219 (3)	C30—C31	1.386 (5)
Ru1—P1	2.2258 (8)	C30—C39	1.435 (5)
Ru1—P2	2.2821 (9)	C31—C32	1.431 (4)
P1—C61	1.843 (3)	C32—C33	1.358 (5)
P1—C71	1.849 (3)	C32—H32A	0.9500
P1—C31	1.864 (3)	C33—C34	1.412 (5)
P2—C51	1.845 (3)	C33—H33A	0.9500
P2—C41	1.847 (3)	C34—C35	1.411 (5)
P2—C21	1.848 (3)	C34—C39	1.430 (4)
O1—C9	1.388 (4)	C35—C36	1.377 (6)
O1—C12	1.422 (5)	C35—H35A	0.9500
O2—C16	1.358 (4)	C36—C37	1.400 (4)
O2—C19	1.433 (4)	C36—H36A	0.9500
N1—H1C	0.88 (4)	C37—C38	1.367 (5)
N1—H1B	0.96 (5)	C37—H37A	0.9500
N1—C1	1.493 (4)	C38—C39	1.414 (5)
N2—H2B	0.76 (3)	C38—H38A	0.9500
N2—H2A	0.95 (4)	C41—C46	1.384 (5)
N2—C2	1.508 (4)	C41—C42	1.414 (5)
C1—C3	1.550 (5)	C42—C43	1.387 (5)
C1—C2	1.564 (4)	C42—H42A	0.9500
C1—H1A	1.0000	C43—C44	1.394 (5)
C2—C13	1.521 (4)	C43—H43A	0.9500
C2—C6	1.525 (4)	C44—C45	1.374 (6)
C3—C4	1.534 (5)	C44—H44A	0.9500
C3—C5	1.548 (5)	C45—C46	1.389 (5)
C3—H3A	1.0000	C45—H45A	0.9500
C4—H4A	0.9800	C46—H46A	0.9500
C4—H4B	0.9800	C51—C52	1.387 (5)
C4—H4C	0.9800	C51—C56	1.391 (5)
C5—H5A	0.9800	C52—C53	1.405 (6)
C5—H5B	0.9800	C52—H52A	0.9500
C5—H5C	0.9800	C53—C54	1.369 (7)
C6—C7	1.386 (5)	C53—H53A	0.9500
C6—C11	1.423 (5)	C54—C55	1.380 (7)
C7—C8	1.397 (5)	C54—H54A	0.9500
C7—H7A	0.9500	C55—C56	1.396 (5)
C8—C9	1.395 (5)	C55—H55A	0.9500
C8—H8A	0.9500	C56—H56A	0.9500
C9—C10	1.390 (5)	C61—C66	1.382 (5)
C10—C11	1.395 (4)	C61—C62	1.406 (5)
C10—H10A	0.9500	C62—C63	1.390 (5)
C12—H12A	0.9800	C62—H62A	0.9500
C12—H12B	0.9800	C63—C64	1.378 (6)
C12—H12C	0.9800	C63—H63A	0.9500
C13—C14	1.387 (4)	C64—C65	1.373 (6)
C13—C18	1.405 (5)	C64—H64A	0.9500
C14—C15	1.395 (5)	C65—C66	1.388 (6)
C14—H14A	0.9500	C65—H65A	0.9500
C15—C16	1.388 (5)	C66—H66A	0.9500
C15—H15A	0.9500	C71—C72	1.387 (5)
C16—C17	1.399 (5)	C71—C76	1.408 (5)
C17—C18	1.379 (5)	C72—C73	1.398 (5)
C17—H17A	0.9500	C72—H72A	0.9500
C18—H18A	0.9500	C73—C74	1.374 (5)
C19—H19A	0.9800	C73—H73A	0.9500
C19—H19B	0.9800	C74—C75	1.389 (6)
C19—H19C	0.9800	C74—H74A	0.9500
C20—C21	1.389 (4)	C75—C76	1.390 (5)
C20—C29	1.437 (4)	C75—H75A	0.9500
C20—C30	1.500 (4)	C76—H76A	0.9500
C21—C22	1.425 (4)	C1S—C2S	1.3900
C22—C23	1.363 (4)	C1S—C6S	1.3900
C22—H22A	0.9500	C1S—H1SA	0.9500
C23—C24	1.407 (5)	C2S—C3S	1.3900
C23—H23A	0.9500	C2S—H2SA	0.9500
C24—C25	1.424 (5)	C3S—C4S	1.3900
C24—C29	1.424 (5)	C3S—H3SA	0.9500
C25—C26	1.340 (5)	C4S—C5S	1.3900
C25—H25A	0.9500	C4S—H4SA	0.9500
C26—C27	1.412 (6)	C5S—C6S	1.3900
C26—H26A	0.9500	C5S—H5SA	0.9500
C27—C28	1.367 (5)	C6S—H6SA	0.9500
			
H1RU—Ru1—C11	87.1 (12)	C26—C25—H25A	119.3
H1RU—Ru1—N1	92.4 (11)	C24—C25—H25A	119.3
C11—Ru1—N1	79.53 (12)	C25—C26—C27	120.0 (3)
H1RU—Ru1—N2	160.8 (12)	C25—C26—H26A	120.0
C11—Ru1—N2	75.93 (12)	C27—C26—H26A	120.0
N1—Ru1—N2	75.76 (10)	C28—C27—C26	120.5 (3)
H1RU—Ru1—P1	88.4 (11)	C28—C27—H27A	119.7
C11—Ru1—P1	92.01 (8)	C26—C27—H27A	119.7
N1—Ru1—P1	171.43 (9)	C27—C28—C29	121.0 (3)
N2—Ru1—P1	101.01 (8)	C27—C28—H28A	119.5
H1RU—Ru1—P2	84.1 (12)	C29—C28—H28A	119.5
C11—Ru1—P2	168.64 (8)	C28—C29—C24	118.2 (3)
N1—Ru1—P2	93.57 (9)	C28—C29—C20	122.4 (3)
N2—Ru1—P2	111.33 (8)	C24—C29—C20	119.4 (3)
P1—Ru1—P2	95.00 (3)	C31—C30—C39	121.2 (3)
C61—P1—C71	103.52 (15)	C31—C30—C20	124.6 (3)
C61—P1—C31	102.32 (16)	C39—C30—C20	114.0 (3)
C71—P1—C31	96.82 (14)	C30—C31—C32	117.8 (3)
C61—P1—Ru1	108.53 (11)	C30—C31—P1	120.4 (2)
C71—P1—Ru1	121.91 (11)	C32—C31—P1	121.9 (3)
C31—P1—Ru1	120.94 (10)	C33—C32—C31	122.0 (3)
C51—P2—C41	102.20 (14)	C33—C32—H32A	119.0
C51—P2—C21	99.88 (15)	C31—C32—H32A	119.0
C41—P2—C21	104.05 (14)	C32—C33—C34	121.3 (3)
C51—P2—Ru1	126.36 (12)	C32—C33—H33A	119.4
C41—P2—Ru1	111.59 (11)	C34—C33—H33A	119.4
C21—P2—Ru1	110.25 (10)	C35—C34—C33	122.5 (3)
C9—O1—C12	117.3 (3)	C35—C34—C39	119.1 (3)
C16—O2—C19	118.0 (3)	C33—C34—C39	118.3 (3)
H1C—N1—H1B	104 (4)	C36—C35—C34	121.3 (3)
H1C—N1—C1	114 (3)	C36—C35—H35A	119.3
H1B—N1—C1	110 (3)	C34—C35—H35A	119.3
H1C—N1—Ru1	97 (3)	C35—C36—C37	119.4 (4)
H1B—N1—Ru1	117 (3)	C35—C36—H36A	120.3
C1—N1—Ru1	114.41 (19)	C37—C36—H36A	120.3
H2B—N2—H2A	102 (3)	C38—C37—C36	120.8 (4)
H2B—N2—C2	110 (3)	C38—C37—H37A	119.6
H2A—N2—C2	113 (2)	C36—C37—H37A	119.6
H2B—N2—Ru1	119 (3)	C37—C38—C39	121.5 (3)
H2A—N2—Ru1	109 (2)	C37—C38—H38A	119.2
C2—N2—Ru1	103.85 (19)	C39—C38—H38A	119.2
N1—C1—C3	113.6 (3)	C38—C39—C34	117.8 (3)
N1—C1—C2	107.3 (2)	C38—C39—C30	123.1 (3)
C3—C1—C2	115.3 (3)	C34—C39—C30	119.1 (3)
N1—C1—H1A	106.7	C46—C41—C42	117.8 (3)
C3—C1—H1A	106.7	C46—C41—P2	122.8 (2)
C2—C1—H1A	106.7	C42—C41—P2	119.0 (2)
N2—C2—C13	109.1 (3)	C43—C42—C41	120.9 (3)
N2—C2—C6	104.8 (2)	C43—C42—H42A	119.6
C13—C2—C6	114.7 (3)	C41—C42—H42A	119.6
N2—C2—C1	104.1 (3)	C42—C43—C44	120.0 (3)
C13—C2—C1	113.8 (2)	C42—C43—H43A	120.0
C6—C2—C1	109.3 (3)	C44—C43—H43A	120.0
C4—C3—C5	109.1 (3)	C45—C44—C43	119.2 (3)
C4—C3—C1	109.7 (3)	C45—C44—H44A	120.4
C5—C3—C1	114.4 (3)	C43—C44—H44A	120.4
C4—C3—H3A	107.8	C44—C45—C46	121.1 (4)
C5—C3—H3A	107.8	C44—C45—H45A	119.5
C1—C3—H3A	107.8	C46—C45—H45A	119.5
C3—C4—H4A	109.5	C41—C46—C45	121.0 (3)
C3—C4—H4B	109.5	C41—C46—H46A	119.5
H4A—C4—H4B	109.5	C45—C46—H46A	119.5
C3—C4—H4C	109.5	C52—C51—C56	118.8 (3)
H4A—C4—H4C	109.5	C52—C51—P2	118.6 (3)
H4B—C4—H4C	109.5	C56—C51—P2	122.2 (3)
C3—C5—H5A	109.5	C51—C52—C53	119.9 (4)
C3—C5—H5B	109.5	C51—C52—H52A	120.1
H5A—C5—H5B	109.5	C53—C52—H52A	120.1
C3—C5—H5C	109.5	C54—C53—C52	120.3 (4)
H5A—C5—H5C	109.5	C54—C53—H53A	119.8
H5B—C5—H5C	109.5	C52—C53—H53A	119.8
C7—C6—C11	121.9 (3)	C53—C54—C55	120.7 (4)
C7—C6—C2	122.7 (3)	C53—C54—H54A	119.7
C11—C6—C2	115.1 (3)	C55—C54—H54A	119.7
C6—C7—C8	121.5 (3)	C54—C55—C56	119.1 (4)
C6—C7—H7A	119.3	C54—C55—H55A	120.4
C8—C7—H7A	119.3	C56—C55—H55A	120.4
C9—C8—C7	117.6 (3)	C51—C56—C55	121.2 (4)
C9—C8—H8A	121.2	C51—C56—H56A	119.4
C7—C8—H8A	121.2	C55—C56—H56A	119.4
O1—C9—C10	116.2 (3)	C66—C61—C62	117.4 (3)
O1—C9—C8	123.3 (3)	C66—C61—P1	122.8 (3)
C10—C9—C8	120.6 (3)	C62—C61—P1	119.1 (3)
C9—C10—C11	123.4 (3)	C63—C62—C61	122.0 (4)
C9—C10—H10A	118.3	C63—C62—H62A	119.0
C11—C10—H10A	118.3	C61—C62—H62A	119.0
C10—C11—C6	115.2 (3)	C64—C63—C62	118.7 (4)
C10—C11—Ru1	130.8 (2)	C64—C63—H63A	120.7
C6—C11—Ru1	113.7 (2)	C62—C63—H63A	120.7
O1—C12—H12A	109.5	C65—C64—C63	120.3 (4)
O1—C12—H12B	109.5	C65—C64—H64A	119.8
H12A—C12—H12B	109.5	C63—C64—H64A	119.8
O1—C12—H12C	109.5	C64—C65—C66	120.8 (4)
H12A—C12—H12C	109.5	C64—C65—H65A	119.6
H12B—C12—H12C	109.5	C66—C65—H65A	119.6
C14—C13—C18	117.3 (3)	C61—C66—C65	120.7 (4)
C14—C13—C2	123.5 (3)	C61—C66—H66A	119.7
C18—C13—C2	119.0 (3)	C65—C66—H66A	119.7
C13—C14—C15	122.2 (3)	C72—C71—C76	118.6 (3)
C13—C14—H14A	118.9	C72—C71—P1	119.6 (2)
C15—C14—H14A	118.9	C76—C71—P1	121.4 (3)
C16—C15—C14	119.3 (3)	C71—C72—C73	120.6 (3)
C16—C15—H15A	120.3	C71—C72—H72A	119.7
C14—C15—H15A	120.3	C73—C72—H72A	119.7
O2—C16—C15	125.0 (3)	C74—C73—C72	120.5 (4)
O2—C16—C17	115.6 (3)	C74—C73—H73A	119.7
C15—C16—C17	119.4 (3)	C72—C73—H73A	119.7
C18—C17—C16	120.3 (3)	C73—C74—C75	119.7 (3)
C18—C17—H17A	119.9	C73—C74—H74A	120.2
C16—C17—H17A	119.9	C75—C74—H74A	120.2
C17—C18—C13	121.3 (3)	C74—C75—C76	120.3 (3)
C17—C18—H18A	119.3	C74—C75—H75A	119.8
C13—C18—H18A	119.3	C76—C75—H75A	119.8
O2—C19—H19A	109.5	C75—C76—C71	120.3 (3)
O2—C19—H19B	109.5	C75—C76—H76A	119.9
H19A—C19—H19B	109.5	C71—C76—H76A	119.9
O2—C19—H19C	109.5	C2S—C1S—C6S	120.0
H19A—C19—H19C	109.5	C2S—C1S—H1SA	120.0
H19B—C19—H19C	109.5	C6S—C1S—H1SA	120.0
C21—C20—C29	120.2 (3)	C1S—C2S—C3S	120.0
C21—C20—C30	123.9 (3)	C1S—C2S—H2SA	120.0
C29—C20—C30	115.6 (3)	C3S—C2S—H2SA	120.0
C20—C21—C22	118.4 (3)	C4S—C3S—C2S	120.0
C20—C21—P2	118.6 (2)	C4S—C3S—H3SA	120.0
C22—C21—P2	122.5 (2)	C2S—C3S—H3SA	120.0
C23—C22—C21	121.8 (3)	C3S—C4S—C5S	120.0
C23—C22—H22A	119.1	C3S—C4S—H4SA	120.0
C21—C22—H22A	119.1	C5S—C4S—H4SA	120.0
C22—C23—C24	121.0 (3)	C6S—C5S—C4S	120.0
C22—C23—H23A	119.5	C6S—C5S—H5SA	120.0
C24—C23—H23A	119.5	C4S—C5S—H5SA	120.0
C23—C24—C25	122.4 (3)	C5S—C6S—C1S	120.0
C23—C24—C29	118.8 (3)	C5S—C6S—H6SA	120.0
C25—C24—C29	118.8 (3)	C1S—C6S—H6SA	120.0
C26—C25—C24	121.5 (3)		
